# Factors influencing maternal microchimerism throughout infancy and its impact on infant T cell immunity

**DOI:** 10.1172/JCI148826

**Published:** 2022-07-01

**Authors:** Christina Balle, Blair Armistead, Agano Kiravu, Xiaochang Song, Anna-Ursula Happel, Angela A. Hoffmann, Sami B. Kanaan, J. Lee Nelson, Clive M. Gray, Heather B. Jaspan, Whitney E. Harrington

**Affiliations:** 1 Division of Immunology, Department of Pathology, Institute of Infectious Disease and Molecular Medicine, University of Cape Town, Cape Town, South Africa.; 2 Seattle Children’s Research Institute, Seattle, Washington, USA.; 3 University of Washington School of Medicine, Seattle, Washington, USA.; 4 Clinical Research Division, Fred Hutchinson Cancer Center, Seattle, Washington, USA.; 5 Department of Pediatrics and; 6 Department of Global Health, University of Washington, Seattle, Washington, USA.

**Keywords:** AIDS/HIV, Immunology, Bacterial vaccines, Obstetrics/gynecology, T cells

## Abstract

Determinants of the acquisition and maintenance of maternal microchimerism (MMc) during infancy and the impact of MMc on infant immune responses are unknown. We examined factors that influence MMc detection and level across infancy and the effect of MMc on T cell responses to bacillus Calmette-Guérin (BCG) vaccination in a cohort of HIV-exposed, uninfected and HIV-unexposed infants in South Africa. MMc was measured in whole blood from 58 infants using a panel of quantitative PCR assays at day 1, and 7, 15, and 36 weeks of life. Infants received BCG at birth, and selected whole blood samples from infancy were stimulated in vitro with BCG and assessed for polyfunctional CD4^+^ T cell responses. MMc was present in most infants across infancy, with levels ranging from 0 to 1,193/100,000 genomic equivalents and was positively impacted by absence of maternal HIV, maternal and infant HLA compatibility, infant female sex, and exclusive breastfeeding. Initiation of maternal antiretroviral therapy prior to pregnancy partially restored MMc level in HIV-exposed, uninfected infants. Birth MMc was associated with an improved polyfunctional CD4^+^ T cell response to BCG. These data emphasize that both maternal and infant factors influence the level of MMc, which may subsequently affect infant T cell responses.

## Graphical abstract

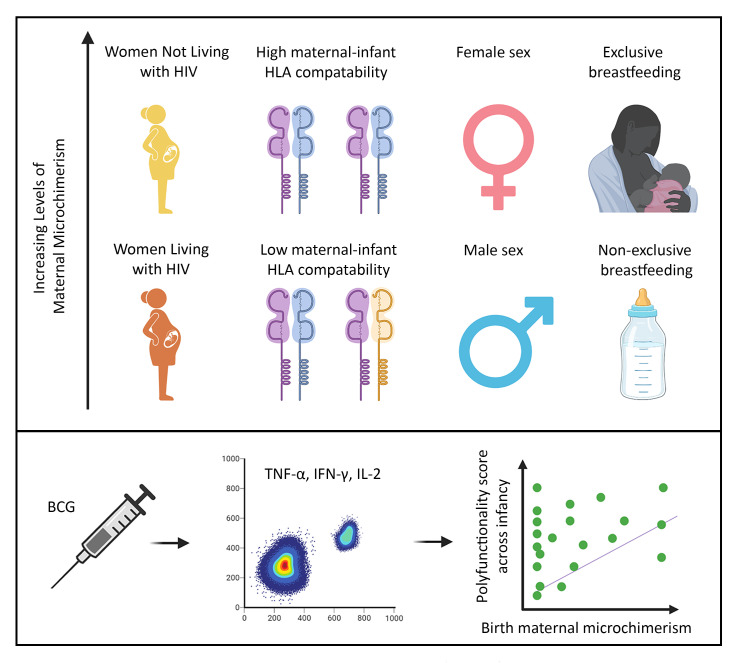

## Introduction

During pregnancy maternal cells are transferred to the fetus and persist in small quantities postnatally, a phenomenon known as maternal microchimerism (MMc). The impact of these maternal cells on the development of the fetal and infant immune system is largely unknown. MMc is acquired as early as the second trimester (1, 2), is found in immune and nonimmune cells throughout the body (3–5), and is maintained into adulthood (3, 6). However, there are limited data on factors that determine the quantity of cells a fetus acquires or those that affect the level of MMc across infancy. In a Tanzanian cohort, we recently demonstrated that placental malaria infection, particularly inflammatory placental malaria, was associated with increased detection and level of cord blood MMc (7). In animal models, breastfeeding has been associated with increased maintenance of in utero–acquired maternal cells, thought to be the product of increased tolerance to noninherited maternal antigens (NIMAs) (8). Further, postnatal transfer of maternal cells to the offspring via breastfeeding has been demonstrated in rodents (9–12) and nonhuman primates (13), suggesting that the maternal graft in the infant may be a composite of cells acquired transplacentally and via the breast milk.

Maternal HIV, even in the absence of vertical transmission, is associated with placental inflammation, including increased expression of inflammatory cell adhesion molecules, CD8^+^ T cell infiltration (14), chorioamnionitis, and deciduitis (15). The impact of maternal HIV on MMc in the infant is unknown. Infants who are HIV exposed but uninfected (iHEU) experience increased morbidity and mortality compared with their HIV-unexposed (iHU) counterparts (16). We have previously shown that iHEUs have altered T cell functionality in response to bacillus Calmette-Guérin (BCG) and superantigens, including lower interferon γ (IFN-γ) and polyfunctional cytokine (IFN-γ, interleukin 2 [IL-2], and IL-17) responses (17), suggesting that altered immunity may contribute to their increased morbidity. Multiple potential mechanisms have been explored to explain such altered immunity, including differences in infant T cell repertoire (18) or changes to infant gut microbiome (19, 20), secondary to an altered in utero environment, including antiretroviral therapy (ART) exposure, feeding modality, or maternal comorbidities (21–24). While 3 studies have investigated the relationship between MMc, HIV, and the risk of vertical transmission (25–27), to the best of our knowledge, no studies to date have examined differences in MMc in iHEU versus iHU as a potential mediator of altered T cell responses in the offspring.

Maternal cells may shape fetal and infant immunity via at least 3 mechanisms. First, prior work has demonstrated an enrichment of maternal cells in antigen-experienced T cells from cord blood (28), suggesting that the offspring may acquire maternal antigen–specific T cells that could have a direct effect when they encounter their cognate antigen in the infant. Second, the acquisition of MMc may indirectly shape the development of the fetal and infant immune system, including influencing the function of myeloid cells, as was recently demonstrated in mice (29). Third, MMc is associated with the development of NIMA-specific regulatory T cells (Tregs), which suppress effector T cell responses to maternal alloantigens (30). Encounter of neoantigen in the presence of such Tregs may induce cross-tolerance, as has been described in transplant biology (8, 31, 32). Supporting the potential for MMc to affect infant outcomes, we have previously found that detection of cord blood MMc in Tanzanian infants was associated with decreased risk of symptomatic malaria (7), and whole blood MMc detection in American children was found to be protective from the later development of asthma (33).

Here, we investigate factors associated with the acquisition and maintenance of MMc in South African infants, including the potential impact of maternal HIV, maternal and infant HLA compatibility, infant sex, and mode of feeding. We further assess the impact of MMc on infant T cell responses directed against BCG, a common neonatal antigen exposure.

## Results

### Cohort characteristics.

We screened 90 mother-infant pairs for a maternal marker. Of these, 58 mothers had a unique marker that could be targeted to detect MMc in the background of the infant. There were no differences in the characteristics of the mother-infant pairs that could or could not be targeted for measurement of MMc (Supplemental Table 1; supplemental material available online with this article; https://doi.org/10.1172/JCI148826DS1). The mean maternal age at delivery was 27 years (standard deviation [SD] 5.1) (Table 1). This was the first pregnancy for 20.7% of the mothers (12 of 58) and the first delivery for 34.5% (20 of 58). The mean estimated gestational age (EGA) at delivery was 39.4 weeks (SD 1.37), and mean infant birth weight was 3,207 grams (SD 416). Just under half of the infants were female (26 of 58, 44.8%). Nearly two-thirds of the mothers were living with HIV (37 of 58, 63.8%), of whom just over half (20 of 37, 54.1%) had received ART prior to conception (early ART), while the remaining mothers initiated ART during pregnancy (late ART) at a mean gestational age of 21 weeks (SD 8.3).

### MMc at birth is modified by HIV exposure, HLA compatibility, and infant sex.

**Figure 1:**
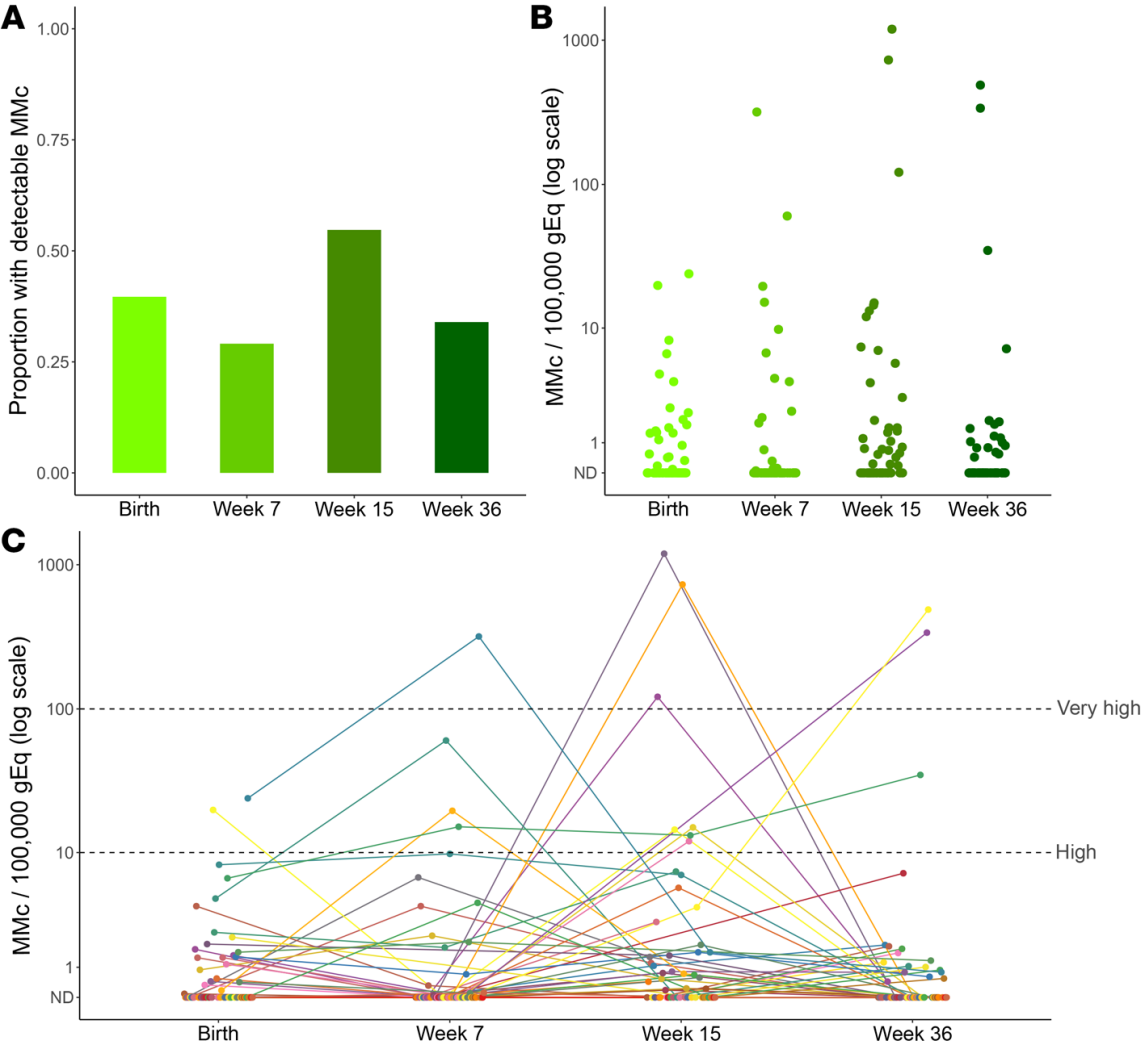
Maternal microchimerism is dynamic and persists throughout infancy. Maternal microchimerism (MMc) expressed as microchimeric equivalents per infant genomic equivalents (gEq) assessed. ND, not detected. (**A**) Proportion of infants with detectable MMc at birth (*n =* 58), week 7 (*n =* 55), week 15 (*n =* 53), and week 36 (*n =* 53) of life. (**B**) Quantitative levels of MMc across infancy. (**C**) Within-infant MMc kinetics. The dotted lines highlight MMc levels above 10/100,000 gEq (high) and above 100/100,000 gEq (very high).

**Figure 2:**
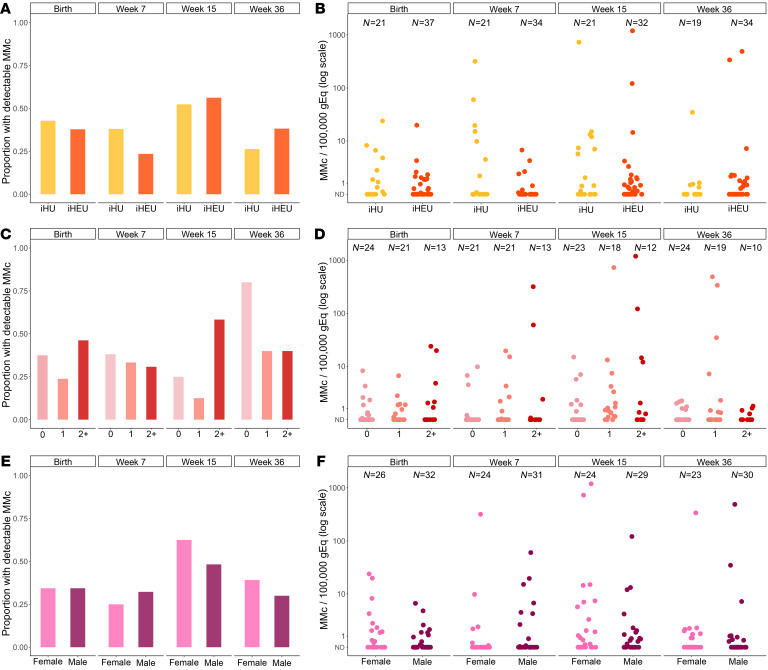
Maternal microchimerism across infancy is associated with HIV exposure, maternal and infant HLA class II compatibility, and infant sex. Maternal microchimerism (MMc) expressed as microchimeric equivalents per infant genomic equivalents (gEq) assessed. ND, not detected; iHU, HIV-unexposed infants; iHEU, HIV-exposed, uninfected infants. Detection of MMc in infants at day 1, week 7, week 15, and week 36 of life by (**A**) HIV exposure, (**C**) maternal and infant HLA class II compatibility (score indicates the number of shared alleles from the mother’s perspective), and (**E**) infant sex. Level of MMc across infancy by (**B**) HIV exposure, (**D**) maternal and infant HLA compatibility, and (**F**) infant sex.

The mean genomic equivalents (gEq) analyzed in whole blood samples collected at day 1 of life (henceforth referred to as birth) was 148,187 (SD 52,867) per sample. Two outcomes were considered for each analysis: (a) the detection of any MMc (no or yes) and (b) the level of MMc. At birth, MMc was detectable in 23 of 58 (39.7%) infants, with levels ranging from 0 to 24/100,000 gEq (Figure 1, A and B). In order to identity factors associated with the detection and quantity of maternal cells at birth, we evaluated the potential impact of maternal HIV status, gravidity, maternal and infant HLA class II compatibility (henceforth referred to as HLA compatibility), EGA, and infant sex. In univariate models, none of the covariates were significantly associated with increased detection of MMc (Figure 2 and Table 2). However, infant female sex was associated with a significantly higher level of MMc (unadjusted detection rate ratio [DRR] 3.71 [95% CI 1.11–12.4], *P =* 0.033; Figure 2F and Table 2) and there were nonsignificantly higher MMc levels in infants with increasing HLA compatibility and lower MMc levels in iHEU and in infants of multigravidae (Figure 2 and Table 2). In multivariate models that included maternal HIV status, gravidity, HLA compatibility, and infant sex, the association between HIV exposure and decreased level of MMc at birth became significant (adjusted DRR 0.34 [95% CI 0.13–0.89], *P =* 0.028), as did the association with increased HLA compatibility (adjusted DRR 1.96 [95% CI 1.16–3.32], *P =* 0.017), while the impact of infant female sex remained significant (adjusted DRR 3.36 [95% CI 1.08–10.5], *P =* 0.037) (Table 2).

### MMc across infancy is dynamic and modified by HIV exposure, HLA compatibility, infant sex, and mode of feeding.

In addition to the birth time point, we also evaluated MMc at week 7, 15, and 36 of life. Across infancy, MMc was detectable at any time point in 44 of 58 (75.9%) infants, with a range of detection of 1 to 3 time points and levels ranging from 0 to 1,193/100,000 gEq (Figure 1, A and B). Within individuals, the level of MMc detected across infancy was dynamic (Figure 1C). Levels of MMc higher than typically detected (>10/100,000 gEq) were identified at at least one time point in 12 of 58 (20.7%) infants, whereas very high MMc (>100/100,000 gEq) was identified at week 7 in 1 infant (318/100,000 gEq), at week 15 in 3 infants (121, 729, and 1,193 per 100,000 gEq), and at week 36 in 2 infants (338 and 488 per 100,000 gEq) (Figure 1C). Across the group, MMc level increased between birth and 15 weeks and then fell at 36 weeks (Figure 1, A and B).

**Figure 3:**
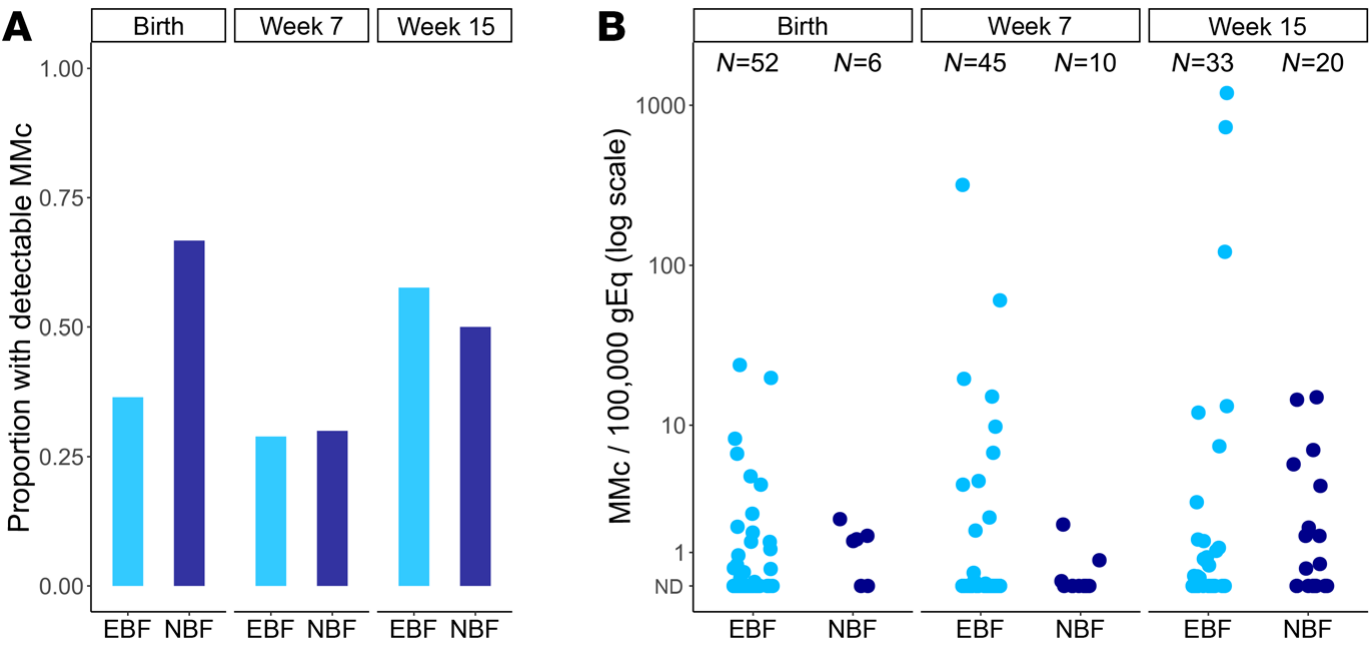
Maternal microchimerism across infancy is associated with mode of feeding. Maternal microchimerism (MMc) expressed as microchimeric equivalents per infant genomic equivalents (gEq) assessed. ND, not detected; EBF, exclusively breastfed; NBF, nonexclusively breastfed. (**A**) Detection of MMc in infants at day 1, week 7, and week 15 of life by mode of feeding. (**B**) Level of MMc across infancy by mode of feeding.

We assessed whether the covariates associated with MMc at birth, as well as the additional covariates of feeding modality and age of the infant, were associated with detection and level of MMc across infancy. Only increased HLA compatibility was significantly associated with increased detection of MMc (adjusted OR 1.54 [95% CI 1.10–2.15], *P =* 0.012; Figure 2C and Table 3). However, HIV exposure was associated with a significantly lower level of MMc (adjusted DRR: 0.37 [95% CI: 0.15–0.94], *P =* 0.036; Figure 2B and Table 3). Higher HLA compatibility was associated with increased level of MMc (adjusted DRR 3.63 [95% CI 2.45–5.39], *P <* 0.001; Figure 2D and Table 3). Compared with male infants, female infants had significantly higher levels of MMc throughout infancy (adjusted DRR 4.56 [95% CI 1.38–15.1], *P =* 0.013; Figure 2, E and F, and Table 3). Exclusive breastfeeding was associated with nonsignificantly higher levels of MMc compared with nonexclusive breastfeeding (adjusted DRR 4.05 [95% CI 0.85–19.4], *P =* 0.080; Table 3). By week 36, only 1 infant remained exclusively breastfed, so to further confirm the associations by mode of feeding, we re-ran our multivariate models, excluding week 36 from the analysis. Exclusive breastfeeding was associated with significantly higher levels of infant MMc in the unadjusted analysis (unadjusted DRR 11.51 [95% CI 3.13–42.33], *P <* 0.001) and nonsignificantly higher levels in the adjusted model (adjusted DRR 2.73 [95% CI 0.92–8.84], *P =* 0.068; Supplemental Table 2 and Figure 3). Finally, older age was significantly associated with higher level of MMc (adjusted DRR per week 1.13 [95% CI 1.07–1.20], *P* < 0.001; Figure 1B and Table 3).

### Initiation of ART prior to conception partially restores MMc in iHEU.

**Figure 4:**
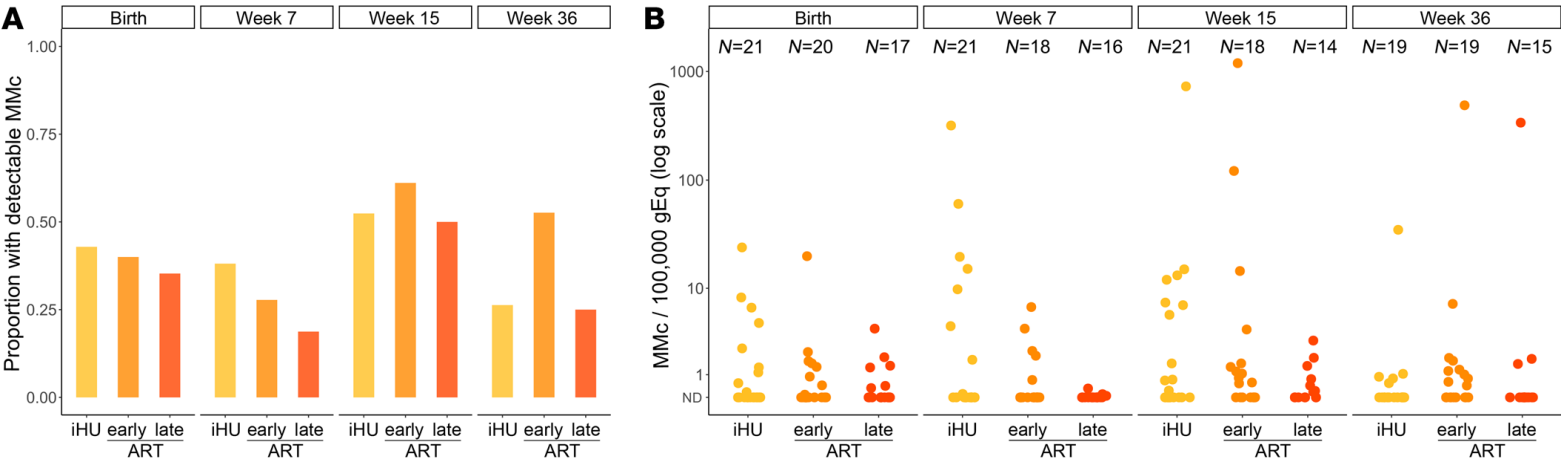
Maternal microchimerism across infancy is associated with duration of maternal antiretroviral therapy (ART). Maternal microchimerism (MMc) expressed as microchimeric equivalents per infant genomic equivalents (gEq) assessed. ND, not detected; iHU, HIV-unexposed infants; early ART, HIV-exposed, uninfected infants whose mothers were on ART before conception; late ART, HIV-exposed, uninfected infants whose mothers initiated ART during pregnancy. (**A**) Detection of MMc in infants at day 1, week 7, week 15, and week 36 of life by HIV exposure and duration of ART. (**B**) Level of MMc across infancy by HIV exposure and duration of ART.

We next assessed whether the timing of ART initiation (preconception or during gestation) altered the effect of maternal HIV on MMc by considering the iHEUs by timing of maternal ART initiation (early or late ART) and repeated the analyses. While both of the HIV-exposed groups had lower levels of MMc compared with the iHUs at birth, this was only significant for infants born to mothers with late ART initiation (Table 4). Similarly, late-ART iHEUs had significantly lower levels of MMc (adjusted DRR 0.14 [95% CI 0.03–0.59], *P =* 0.007; Figure 4B and Table 4) across infancy as compared with iHUs, while early-ART iHEUs had MMc levels similar to those of the iHU infants (Figure 4 and Table 4). When the 2 groups of iHEUs were compared to each other, there was no difference in detection or level of birth MMc. However, there was an increased level (adjusted DRR 4.81 [95% CI 1.00–23.01], *P =* 0.050) of MMc among early- versus late-ART iHEUs across infancy. Collectively, these data indicate that longer duration of maternal ART (from preconception) may partially restore MMc levels in iHEU.

### MMc at birth is associated with polyfunctional CD4^+^ T cell responses to BCG.

**Figure 5:**
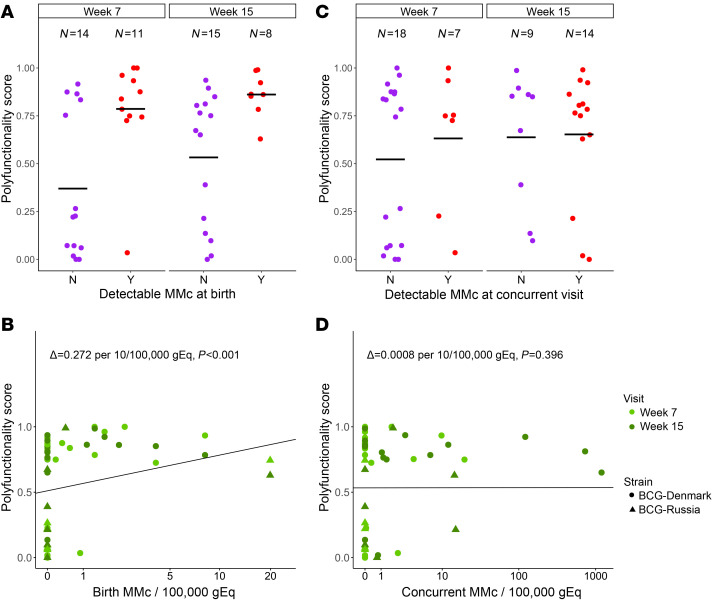
Maternal microchimerism at birth is positively associated with T cell responses to BCG vaccination. (**A**) Polyfunctionality score (PFS) at week 7 and week 15 of life by detection of maternal microchimerism (MMc) at birth (N = no [purple], Y = yes [red]). (**B**) Association between PFS at week 7 (light green) and week 15 (green) of life and level of MMc per 100,000 genomic equivalents (gEq) at birth. Delta represents the adjusted effect size per 10/100,000 gEq. (**C**) PFS at week 7 and week 15 of life by detection of MMc at the concurrent time point (N = no [purple], Y = yes [red]). (**D**) Association between PFS at week 7 (light green) and week 15 (green) of life and level of MMc per 100,000 gEq at the concurrent time point. Delta represents the adjusted effect size per 10/100,000 gEq. Horizontal black lines in **A** and **C** indicate mean values, and black lines in **B** and **D** indicate best fit lines.

Using previously measured BCG vaccine response data from this cohort (34), we sought to determine whether the detection or level of MMc at time of BCG vaccination (birth) would predict BCG-specific T cell responses during early infancy (week 7 and week 15). We used the COMPASS polyfunctional score (PFS), a clinically validated tool for assessing polyfunctional T cell function (35) to measure CD4^+^ T cell responses in 33 infants across 48 samples and adjusted for BCG strain (Danish versus Russian), HIV exposure, and infant age in our analyses. Both detection (adjusted coefficient 0.340, *P <* 0.001; Figure 5A) and level of MMc (adjusted coefficient per 10/100,000 gEq 0.272, *P <* 0.001; Figure 5B) at birth were positively associated with the PFS. In contrast, concurrent detection or level of MMc in the infant blood at week 7 or 15 was not associated with polyfunctionality of BCG response at those time points (Figure 5, C and D). Similar associations were found for the functionality score (FS) (Supplemental Figure 1).

To evaluate whether maternal BCG-responsive T cells directly contributed to the polyfunctional T cell responses to BCG detected in infants, we identified infants who had detectable MMc at birth, a PFS of greater than 0.6, and available peripheral blood mononuclear cells (PBMCs) for functional evaluation (*n =* 4). Infant PBMCs from these time points were stimulated with BCG culture filtrate proteins, followed by intracellular cytokine staining. T cells were sorted into polyfunctional, monofunctional, or nonresponding populations, and we measured MMc level in each population by qPCR. While we were able to detect an enrichment of maternal T cells in the polyfunctional population from 1 iHU, they remained the minority of the responding population, and no MMc was detected in the polyfunctional cells in the other 3 infants (Table 5), indicating that maternal BCG-specific T cells did not directly contribute to the polyfunctional T cell response to BCG in these infants.

## Discussion

This study is the first to our knowledge to measure and compare MMc acquisition and maintenance in iHEUs and iHUs during the first year of life. We observed MMc at least at one time point in the majority of infants in the cohort, with high and very high levels observed in some infants at the later time points. At birth, lack of HIV exposure, increased HLA compatibility, and female infant sex were positively associated with MMc. Across infancy, MMc increased up to a peak at 15 weeks of age and was positively associated with lack of HIV exposure, increased HLA compatibility, female infant sex, and exclusive breastfeeding. Finally, MMc at birth was associated with increased polyfunctional T cell response to BCG later in life, suggesting that these maternal cells may have functional consequence for infant immune responses.

We observed a lower level of MMc in iHEUs compared with iHUs in this cohort. Limited work in human cord blood from term pregnancies found the highest level of MMc in the antigen-experienced T cell subset (28). In mice, the majority of breast milk cells undergoing transepithelial migration were CD4^+^ and CD8^+^ T cells (11). Thus, we hypothesize that the reduction in MMc observed in iHEUs may be due to a reduction in CD4^+^ T cells in the mothers, leading to an overall reduced trafficking of these cells to the fetus and infant. Alternatively, HIV is associated with systemic immune dysregulation, chronic antigen-presenting cell activation (36, 37), and placental inflammation (14, 15), which may lead to altered maternal-fetal tolerance and increased rejection of the maternal graft. The reduced MMc conferred by HIV exposure may contribute to abnormal immune responses and increased infectious morbidity in exposed offspring (16, 38, 39).

Interestingly, we observed that iHEUs born to mothers who initiated ART before conception had higher levels of MMc than those born to mothers who initiated ART during the current pregnancy, and that in the former, levels were comparable to those in iHUs. Early ART may normalize levels of MMc to those seen in iHUs through the restoration of maternal CD4^+^ T cells and/or improvement in systemic immune dysregulation or placental inflammation. These potential mechanisms should be examined in future studies. As MMc acquisition has been observed as early as the beginning of the second trimester (1, 2), initiating ARTs during a later antenatal visit may fail to restore normal MMc levels in the offspring.

In this cohort, increased HLA class II compatibility between mother and infant was associated with increased levels of MMc. This observation is consistent with earlier work finding an association between MMc and maternal compatibility at the HLA class II DRB1 and DQB1 loci (40) as well as animal models (41). HLAs play a critical role in immune regulation (42, 43) and increasing degree of maternal and infant HLA compatibility may lead to increased tolerance of the maternal graft.

There are limited data comparing MMc in female and male offspring. Here, we found that female infants had higher levels of MMc than male infants. This observation is consistent with prior studies comparing children aged 1 to 7 years born to asthmatic and nonasthmatic mothers where MMc detection was higher in daughters (24.3%) compared with sons (16.9%; ref. 29). Increased accumulation of MMc in female compared with male offspring may offer protection against complications in next-generation pregnancies, as shown in mice (44). Further, differences in MMc levels between females and males during early infancy could potentially lead to distinct effects on infant immune development, including differences in vaccine responses and disease susceptibility (40). Additional studies are needed to elucidate the potential mechanism behind this finding.

Our results suggest that exclusive breastfeeding may be associated with higher levels of MMc across infancy compared with nonexclusive breastfeeding. Breastfeeding plays a major role in protection from infection in infants through transfer of antibodies and other immunomodulatory components (45–48). Recent work has demonstrated that breast milk–derived IgA can mediate cross-generational effects on Treg development in the offspring, emphasizing the potential for non–genetically encoded heritability (49). Recent data from animal models has demonstrated that breast milk–derived maternal cells (10–13), including pathogen-experienced T cells (9), can be detected in the offspring. Our study raises the possibility that postnatal MMc may be acquired via breast milk in humans (50), which may be more pronounced during the early stages of lactation when breast milk cells are more abundant (51) and infant gut permeability the highest (52). Alternatively, the increased levels of MMc in exclusively breastfed infants could be due to increased NIMA-specific tolerance leading to maintenance of in utero–acquired MMc (8, 30, 44, 53).

We observed that at the population level MMc increased with infant age, peaking at week 15 followed by a decline at week 36. At this time point, only 1 infant remained exclusively breastfed. The reduction in breastfeeding or the introduction of solid foods or formula could lead to a loss in MMc due to increased intestinal or systemic inflammation leading to rejection of maternal cells or due to the lack of continued transfer of maternal breast milk cells. Distinguishing between these possibilities is challenging in humans but may be possible in future cohorts through improved characterization of the differences between breast milk T cells and the maternal peripheral T cell repertoire. Of note, prior work has found that transplacentally acquired maternal cells traffic into fetal lymph nodes, whereas animal models suggest that breast milk–derived maternal cells traffic to the liver and lung (12, 13), with possible differential effects on both priming and recall responses in the infant.

While the level of MMc we identified in most infants at any time point was similar to prior work (0 to 10/100,000 gEq) (5, 54), in a proportion of infants we observed very high levels of MMc at select time points, such that up to 1% of cells appeared to be of maternal origin, higher than typically described. This observation raises many essential follow-up questions about the origin, phenotype, and function of these cells. Specifically, we hypothesize that these discrete high levels of MMc may represent maternal cell proliferation in response to an exogenous stimulus (e.g., infection or vaccination in the infant), which should be evaluated in future studies.

In order to understand the functional consequence of MMc, we asked whether birth or concurrent MMc was associated with polyfunctionality of CD4^+^ T cell responses to BCG in the infant. Polyfunctional T cells may be indicative of the quality of immune responses to vaccines and have been associated with better clinical prognosis during tuberculosis (TB), HIV, and other infections as compared with the absolute magnitude of T cell responses (35, 55–59). We were particularly motivated to assess this outcome due to prior data suggesting that HIV-exposed infants may mount attenuated immune responses to BCG (17, 60, 61). We identified a positive correlation between birth, but not concurrent, MMc and the polyfunctionality of the T cell response to BCG during early infancy. Since our recruiting site was in Khayelitsha, Western Cape, South Africa, with one of the highest TB rates globally (62, 63), we hypothesized that the polyfunctional cells we detected in infants might reflect maternal mycobacteria-responsive T cells. Notably, we did not find evidence that this was the case, suggesting that maternal cells present at birth may play an indirect role in shaping infants’ immune responses to BCG. This alternative possibility is consistent with prior work in a murine model of MMc, which found that MMc modulates the development of the myeloid lineage, subsequently enhancing neonatal immunity to pathogenic challenge (29). These observations suggest that lower MMc in iHEUs at birth may contribute to altered BCG vaccine responses in these infants.

Our study has a number of limitations. First, our samples were limited to mother-infant pairs where there was a nonshared, noninherited maternal marker that could be targeted with our assays. However, there were no differences in the characteristics of the mother-infant pairs that could or could not be targeted for measurement of MMc. Secondly, at the time of recruitment, HIV viral loads and CD4^+^ counts were not routinely checked as part of prenatal care, so the relationship between viral load, CD4^+^ count, and MMc could not be evaluated; these potential determinants of maternal graft size and composition should be investigated in future studies. Due to robust prenatal screening and high maternal ART coverage, there was only 1 infant infected with HIV in the Innate Factors Associated with Nursing Transmission (InFANT) cohort, and very few in the Western Cape in South Africa, precluding our ability to study this group. Furthermore, HIV infection and ART exposure is associated with higher rates of preterm birth (64–67); however, premature infants were not eligible to enroll in this study, and we may have had different findings had they been included. Finally, BCG response data were only available for a subset of the cohort, although there were no appreciable differences between those infants with and without data available.

To our knowledge, this is the first study to assess MMc in infants across the first year of life and relate their levels to infant T cell response to vaccination. Our findings highlight the as yet largely unexplored impact of this cross-generation graft of maternal cells on infant immunity. Furthermore, our findings provide insight into an additional potential mechanism that may contribute to altered immune responses in iHEUs and the importance of maternal treatment to affect infant outcome. Future work should investigate the cellular phenotype and antigen specificity of these inherited maternal cells.

## Methods

### Cohort.

Data and samples were utilized from the InFANT study, a prospective birth cohort study conducted in Khayelitsha, Cape Town, South Africa, funded by the Canadian Institute for Health Research (co-PIs CM Gray and HB Jaspan). Mother-infant pairs were recruited at the Midwifery Obstetric Unit at Site B in Khayelitsha, Cape Town, South Africa, from March 2014 to March 2018. Infants were followed from birth, at day 4 to 7, and at weeks 7, 15, and 36 of life. Voluntary counseling and HIV testing was done at the time of antenatal care registration. Both pregnant women living with HIV (WLHIV) and women not living with HIV were eligible for the study. Pregnant WLWH and their infants were provided with ART according to the current country-specific guidelines (68). Exclusive breastfeeding was advised to all mothers from delivery to at least 6 months. Infants born before 36 weeks of gestation and with birth weights lower than 2.4 kg were ineligible for the study. Further exclusion criteria included pregnancy or delivery complications as previously described (69). All infants who were classified as iHEU were confirmed as HIV negative by PCR at delivery and at later time points according to prevention of vertical transmission guidelines (68). Routine vaccines were given to all infants according to the WHO’s Expanded Program on Immunization (EPI). Infants received intradermal Danish BCG strain (1331, Statens Serum Institute) from April 2013 to January 2016 and thereafter Russian strain (BCG-I Moscow, Serum Institute of India). Both strains were given at 2 × 10^5^ CFU/dose at birth. Mother-infant pairs were included in the present study based on available paired maternal and infant samples (*n =* 90) and informative typing for a maternal allele with an assay available (*n =* 58).

### Genomic DNA extraction.

Whole blood samples were collected on day 1 (birth) and weeks 7, 15, and 36 of life into sodium-heparin tubes. A 200-μL aliquot of the whole blood was lysed using FACS lysis buffer and subsequently fixed and stored at –80°C until genomic DNA (gDNA) extraction. The gDNA was extracted from fixed whole blood (WBF) samples from infants and mothers using QiaAMP DNA Mini Kits (Qiagen), with slight modifications to the manufacturer’s protocol. In brief, WBF samples were thawed and centrifuged for 2 minutes to pellet cells. The supernatant was discarded and the pellet resuspended in 180 μL Qiagen Buffer ATL and 20 μL proteinase K followed by incubation at 56°C for 1 hour. This was followed by addition of 200 μL Buffer AL and 200 μL 100% ethanol and the entire sample lysate was transferred onto the QIAamp MinElute column. The gDNA was eluted using 200 μL molecular grade water, which was allowed to incubate in the column for 10 minutes at room temperature to increase DNA yield.

### Identification of maternal polymorphisms and microchimerism evaluation.

Genomic DNA from maternal and infant granulocytes collected at week 36 was genotyped at HLA class II loci DRB1, DQA1, and DQB1 by direct sequencing (Scisco Genetics). Noninherited, nonshared HLA polymorphisms were identified that could be used to evaluate MMc (3, 70). Mother-infant pairs with noninformative HLA typing were typed at 4 non-HLA loci: *GSTT1*, *ATIII*, *TG*, and *TNN*, targeting insertion/deletion polymorphisms (28). The maternal polymorphism identified for each mother-infant pair was selectively amplified from WBF gDNA using a panel of previously developed qPCR assays (28, 70, 71). The limit of detection of each of the qPCR assays is 1 target gEq in up to 60,000 background gEq per reaction well (72). DNA from each WBF was distributed across multiple reaction wells, targeting a total gEq of 120,000 per sample. A calibration curve for the polymorphism-specific assay was included to quantify the amount of MMc in each well, and the microchimeric gEq was summed across all tested wells. Total gEq tested for each sample was determined by targeting the nonpolymorphic β-globin gene, and a β-globin calibration curve (human genomic DNA, Promega) was concurrently evaluated on each plate. Level of MMc is presented as the total microchimeric gEq proportional to the total gEq tested for each sample.

### Maternal and infant HLA class II compatibility score.

To analyze the degree of HLA class II relatedness for each mother-infant pair, we generated a scoring system based on whether the infant’s paternal allele at each locus (DPA1, DPB1, DQA1, DQB1, DRB1) was a match to self from the mother’s perspective. A score of 0 at a single locus signified no match between the infant’s paternally inherited allele and either of the mother’s 2 alleles; a score of 1 signified a match between the infant’s paternally inherited allele and at least one of the mother’s alleles at that locus. For each pair, we summed the score at each locus to give an HLA compatibility score from 0 to 5 (0 signifies no HLA relatedness between infant’s paternally derived allele and mother’s alleles at any loci; 5 signifies a match at each locus between the infant’s paternally derived allele and at least one of the mother’s alleles).

### Whole blood BCG stimulation assay.

A 12-hour whole blood assay was used to evaluate vaccine responses as previously described (73). Briefly, 250 μL of anticoagulated blood was incubated with BCG, media, or phytohemagglutinin at 37°C within 1 hour of blood draw. After 5 hours, brefeldin A (Sigma-Aldrich) was added and incubated for an additional 7 hours. Thereafter, red blood cells were lysed followed by washing and staining with LIVE/DEAD fixable Violet stain (ViViD, Thermo Fisher Scientific). The cells were cryopreserved in liquid nitrogen.

### Cell staining, antibodies, and flow cytometry for BCG whole blood assay.

Staining and flow cytometry were conducted as previously described (34). In brief, batched stored samples were thawed quickly and washed, incubated in BD PermWash, and then stained with the antibody cocktail mix. After incubation, cells were washed and then resuspended in PBS for cell acquisition using a BD LSRII flow cytometer (SORF model). The following monoclonal antibody–fluorochrome conjugates were used: IL-2-R–phycoerythrin (PE), CD8-V500, IFN-γ–Alexa Fluor 700, TNF-α–PE-Cy7, Ki67–fluorescein isothiocyanate (FITC), all from BD; CD27–PE-Cy5, HLA-DR–allophycocyanin-Cy7 (APC-Cy7), CD3-BV650 (BioLegend); CD4–PE-Cy5.5 (Invitrogen); and CD45RA–PE-Texas Red-X (Beckman Coulter). A minimum of 50,000 ViViD-negative (viable) CD3^+^ events were collected using BD FACSDiva v6 software. Postacquisition compensation and analysis was performed in FlowJo version 9 (FlowJo, LLC). Supplemental Figure 2 shows the gating strategy employed. Measurable response to BCG was characterized by the polyfunctionality of the CD3^+^CD4^+^ T cell response assessed by analyzing permutations of TNF-α, IL-2, and IFN-γ expression after stimulation using combinatorial polyfunctionality analysis of antigen-specific T cell subsets (COMPASS) (35). PFS and FS, which summarize the functional profile of each subject, were calculated from posterior probabilities as described previously (35).

### Stimulation, cell staining, and cell sorting for detection of maternal mycobacteria-specific T cells.

PBMCs were isolated via density gradient centrifugation from infant whole blood. Available PBMCs from infants who had detectible MMc at birth (in whole blood) and a COMPASS PFS of greater than 0.6 at the concurrent time point were selected for stimulation with BCG culture filtrate proteins. PBMCs were thawed and treated with BD FastTimmune anti-CD28/anti-CD49d costimulatory antibodies (1:100, catalog 347690), brefeldin A (1:1000, BioLegend), and BD GolgiStop (1:1500). PBMCs were then stimulated with BCG culture filtrate proteins (100 μg/mL, BEI Resources), media, or PMA (0.05 μg/mL, MilliporeSigma) and ionomycin (1 μM, MilliporeSigma) and incubated at 37°C, 5% CO_2_ for 6 hours, upon which the reaction was stopped with the addition of EDTA (2 mM). Cells were treated with human Fc block (BD, 1:200) and then stained with LIVE/DEAD Fixable Aqua (Thermo Fisher Scientific) and the following extracellular monoclonal antibody–fluorochrome conjugates: CD3–PE-Cy7 (BioLegend, catalog 344816), CD4-BV421 (BioLegend, catalog 300532), CD8–PerCP-Cy5.5 (BioLegend, catalog 301032), CCR7-BV785 (BioLegend, catalog 353230), and CD45RO–PE-CF594 (BD, catalog 562299) or CD45RO-APC (BioLegend, catalog 304210). Cells were washed and then resuspended in Cyto-Fast Fix/Perm Buffer (BioLegend), followed by washing in Cyto-Fast Perm Wash solution (BioLegend). Cells were then stained with intracellular monoclonal antibody–fluorochrome conjugates IL-2–PE (BioLegend, catalog 500307), TNF-α–FITC (BD, catalog 554512), and IFN-γ–BV605 (BioLegend, catalog 506542). Cells were washed, resuspended in cell staining buffer, and run on a BD FACSAria II cell sorter with single-stained AbC Total Antibody or ArC Amine Reactive Compensation Beads as compensation controls. To sort BCG-stimulated T cells into a polyfunctional population (positive for 2 or 3 of the cytokines IL-2, TNF-α, and IFN-γ), a monofunctional population (positive for 1 cytokine), and a nonresponsive population (negative for the 3 cytokines), cells were gated according to the scheme in Supplemental Figure 3. Genomic DNA was isolated from each sorted population, and MMc was quantified using qPCR assays targeting a maternal marker, as described above.

### Statistics.

Statistical analysis was performed using Stata 14 software (StataCorp LP) and R (version 4.0.3). Detection of MMc at birth was evaluated by logistic regression with adjustment for the total number of gEq tested for each subject. Level of MMc at birth was evaluated with negative binomial regression, accounting for the number of microchimeric gEq detected as well as the total number of gEq assessed in each sample (7, 28, 71, 72, 74, 75). This approach accounts for the non-normal distribution of MMc data as well as the large number of zeros (74). The output of this model is DRR, which can be interpreted as “X number of microchimeric gEq in group A for every one microchimeric gEq in group B.” Each covariate of interest was first tested individually in univariate models. For the adjusted model, we included HIV exposure and mode of feeding a priori, as they were primary covariates of interest and included other covariates if they were associated with either detection or level of MMc at a *P* value of less than 0.1 (infant sex, maternal and infant HLA class II compatibility score, and gravidity).

Detection of MMc across infancy was assessed using generalized estimating equation (GEE) models clustered by individual, a binomial outcome structure, and an independent correlation matrix. Level of MMc across infancy was assessed using GEE models clustered by individual, a negative binomial outcome structure, and an independent correlation matrix. In addition to the covariates mentioned above, mode of feeding and infant age in weeks were considered for inclusion in the adjusted models.

The effect of MMc on the polyfunctionality of BCG vaccine responses (PFS) were assessed using GEE models clustered by individual, a gaussian outcome structure, and an independent correlation matrix. We have previously shown that iHEU infants have altered T cell functionality in response to BCG (17) and that the type of BCG strain used for vaccination contributes strongly to the magnitude and polyfunctionality of vaccine response elicited in infants (34). We therefore adjusted for BCG strain, HIV exposure, and infant age in our analyses.

### Study approval.

The study was approved by the University of Cape Town Human Research Ethics Committee (Rec ref 285/2012) and the Institutional Review Board of Seattle Children’s Hospital (Protocol 15690). All mothers in the study were of consenting age and provided written informed consent for their and their infant’s participation.

## Author contributions

CB, BA, HBJ, SBK, CMG, JLN, and WEH conceived and designed the experiments. HBJ and CMG designed and recruited the InFANT cohort. CB, BA, AK, AAH, and AUH processed samples and performed wet lab experiments. CB, WEH, XS, and AK analyzed the data. CB, HBJ, and WEH wrote the original draft of the manuscript. All authors reviewed and edited the manuscript.

## Supplementary Material

Supplemental data

## Figures and Tables

**Table 5:**
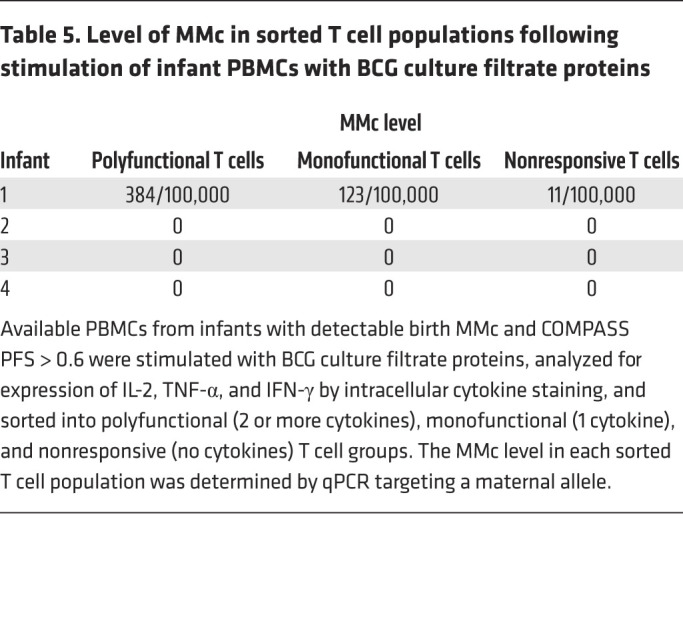
Level of MMc in sorted T cell populations following stimulation of infant PBMCs with BCG culture filtrate proteins

**Table 4:**
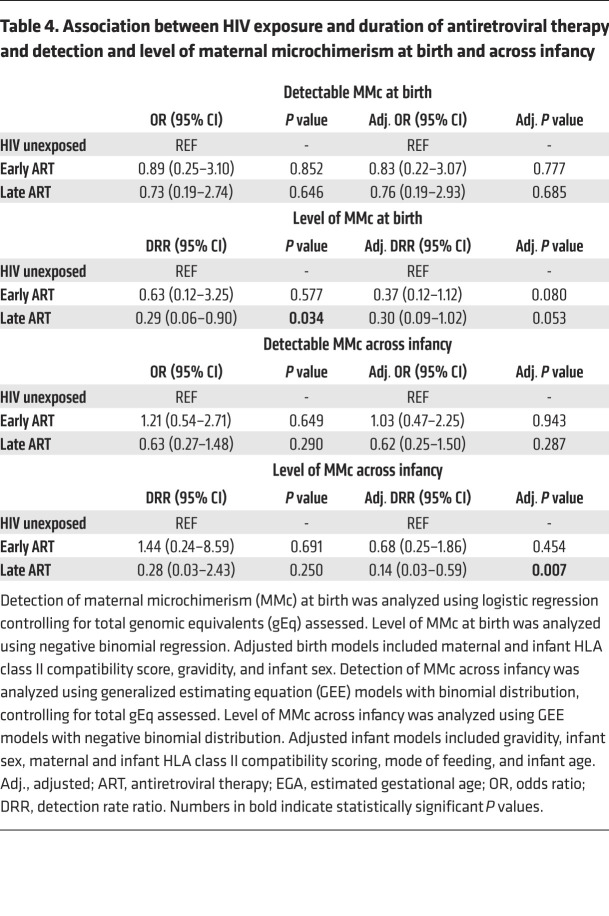
Association between HIV exposure and duration of antiretroviral therapy and detection and level of maternal microchimerism at birth and across infancy

**Table 3:**
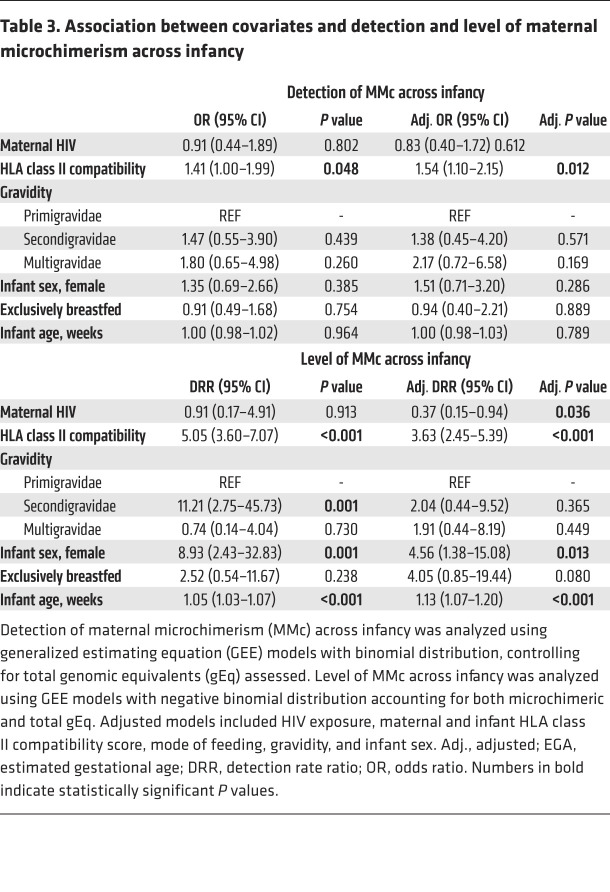
Association between covariates and detection and level of maternal microchimerism across infancy

**Table 2:**
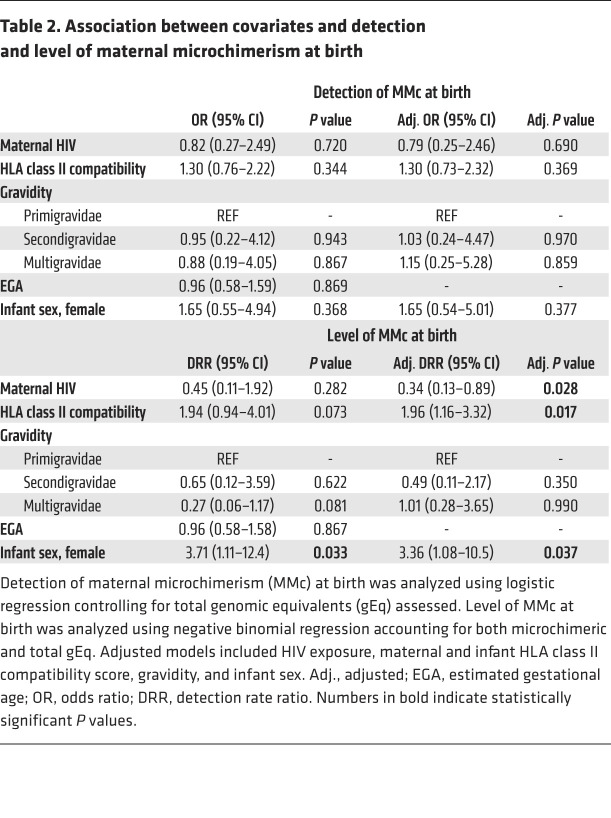
Association between covariates and detection

**Table 1:**
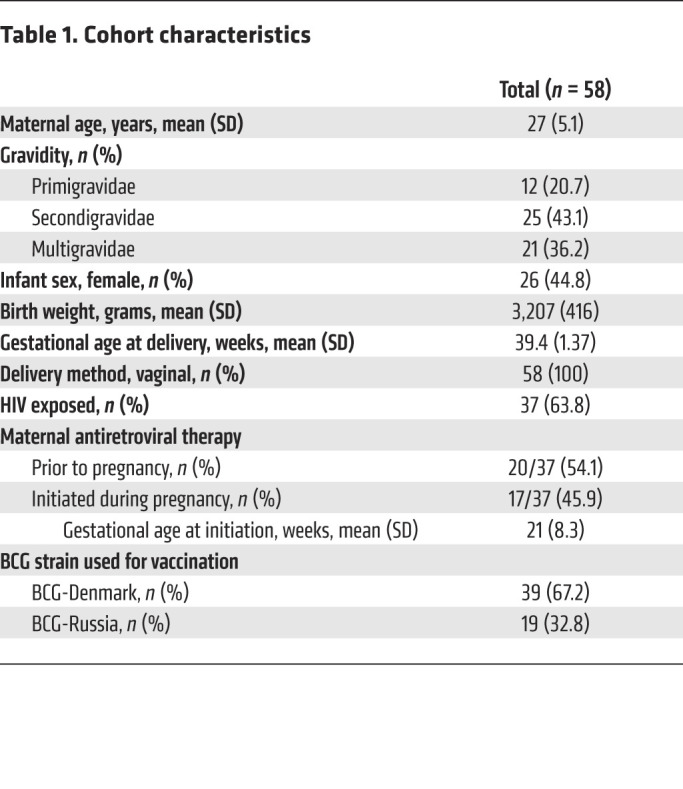
Cohort characteristics

## Version 1 05/12/2022

In-Press Preview

## Version 2 07/01/2022

Electronic publication

## References

[1] Lo ES (1998). Transfer of nucleated maternal cells into fetal circulation during the second trimester of pregnancy. Br J Haematol.

[2] Jonsson AM (2008). Maternal microchimerism in human fetal tissues. Am J Obstet Gynecol.

[3] Loubiere LS (2006). Maternal microchimerism in healthy adults in lymphocytes, monocyte/macrophages and NK cells. Lab Invest.

[4] Hall JM (1995). Detection of maternal cells in human umbilical cord blood using fluorescence in situ hybridization. Blood.

[5] Nelson JL (2012). The otherness of self: microchimerism in health and disease. Trends Immunol.

[6] Maloney S (1999). Microchimerism of maternal origin persists into adult life. J Clin Invest.

[7] Harrington WE (2017). Maternal microchimerism predicts increased infection but decreased disease due to plasmodium falciparum during early childhood. J Infect Dis.

[8] Dutta P (2009). Microchimerism is strongly correlated with tolerance to noninherited maternal antigens in mice. Blood.

[9] Darby MG (2019). Pre-conception maternal helminth infection transfers via nursing long-lasting cellular immunity against helminths to offspring. Sci Adv.

[10] Aydın MŞ (2018). Transfer and integration of breast milk stem cells to the brain of suckling pups. Sci Rep.

[11] Ma LJ (2008). Trans-epithelial immune cell transfer during suckling modulates delayed-type hypersensitivity in recipients as a function of gender. PLoS One.

[12] Zhou L (2000). Two independent pathways of maternal cell transmission to offspring: through placenta during pregnancy and by breast-feeding after birth. Immunology.

[13] Jain L (1989). In vivo distribution of human milk leucocytes after ingestion by newborn baboons. Arch Dis Child.

[14] Baurakiades E (2011). Histomorphometric and immunohistochemical analysis of infectious agents, T-cell subpopulations and inflammatory adhesion molecules in placentas from HIV-seropositive pregnant women. Diagn Pathol.

[15] Schwartz DA (2000). Placental abnormalities associated with human immunodeficiency virus type 1 infection and perinatal transmission in Bangkok, Thailand. J Infect Dis.

[16] Marinda E (2007). Child mortality according to maternal and infant HIV status in Zimbabwe. Pediatr Infect Dis J.

[17] Kidzeru EB (2014). In-utero exposure to maternal HIV infection alters T-cell immune responses to vaccination in HIV-uninfected infants. AIDS.

[18] Gabriel B (2019). Analysis of the TCR repertoire in HIV-exposed but uninfected infants. Sci Rep.

[19] Bender JM (2016). Maternal HIV infection influences the microbiome of HIV-uninfected infants. Sci Transl Med.

[20] Machiavelli A (2019). The impact of in utero HIV exposure on gut microbiota, inflammation, and microbial translocation. Gut Microbes.

[21] Bork KA (2014). Morbidity in relation to feeding mode in African HIV-exposed, uninfected infants during the first 6 mo of life: the Kesho Bora study. Am J Clin Nutr.

[22] Ramokolo V (2017). In utero ART exposure and birth and early growth outcomes among HIV-exposed uninfected infants attending immunization services: results from national PMTCT surveillance, South Africa. Open Forum Infect Dis.

[23] Shapiro RL (2007). Infant morbidity, mortality, and breast milk immunologic profiles among breast-feeding HIV-infected and HIV-uninfected women in Botswana. J Infect Dis.

[24] Mallampati D (2018). Optimal breastfeeding durations for HIV-exposed infants: the impact of maternal ART use, infant mortality and replacement feeding risk. J Int AIDS Soc.

[25] Biggar RJ (2008). The role of transplacental microtransfusions of maternal lymphocytes in HIV transmission to newborns. AIDS.

[26] Lee T-H (2010). The role of transplacental microtransfusions of maternal lymphocytes in in utero HIV transmission. J Acquir Immune Defic Syndr.

[27] Kwiek JJ (2008). Maternal-fetal DNA admixture is associated with intrapartum mother-to-child transmission of HIV-1 in Blantyre, Malawi. J Infect Dis.

[28] Kanaan SB (2017). Maternal microchimerism is prevalent in cord blood in memory T cells and other cell subsets, and persists post-transplant. Oncoimmunology.

[29] Stelzer IA (2021). Vertically transferred maternal immune cells promote neonatal immunity against early life infections. Nat Commun.

[30] Mold JE (2008). Maternal alloantigens promote the development of tolerogenic fetal regulatory T cells in utero. Science.

[31] Bemelman F (1998). Bone marrow transplantation induces either clonal deletion or infectious tolerance depending on the dose. J Immunol.

[32] Qin S (1993). “Infectious” transplantation tolerance. Science.

[33] Thompson EE (2013). Maternal microchimerism protects against the development of asthma. J Allergy Clin Immunol.

[34] Kiravu A (2019). Bacille Calmette-Guérin vaccine strain modulates the ontogeny of both mycobacterial-specific and heterologous T cell immunity to vaccination in infants. Front Immunol.

[35] Lin L (2015). COMPASS identifies T-cell subsets correlated with clinical outcomes. Nat Biotechnol.

[36] Stiksrud B (2019). Activated dendritic cells and monocytes in HIV immunological nonresponders: HIV-induced interferon-inducible protein-10 correlates with low future CD4^+^ recovery. AIDS.

[37] Sabado RL (2010). Evidence of dysregulation of dendritic cells in primary HIV infection. Blood.

[38] Brennan AT (2016). A meta-analysis assessing all-cause mortality in HIV-exposed uninfected compared with HIV-unexposed uninfected infants and children. AIDS.

[39] Evans C (2016). HIV-exposed, uninfected infants: new global challenges in the era of paediatric HIV elimination. Lancet Infect Dis.

[40] Berry SM (2004). Association of maternal histocompatibility at class II HLA loci with maternal microchimerism in the fetus. Pediatr Res.

[41] Kaplan J, Land S (2005). Influence of maternal-fetal histocompatibility and MHC zygosity on maternal microchimerism. J Immunol.

[42] Papúchová H (2019). The dual role of HLA-C in tolerance and immunity at the maternal-fetal interface. Front Immunol.

[43] Crux NB, Elahi S (2017). Human leukocyte antigen (HLA) and immune regulation: how do classical and non-classical HLA alleles modulate immune response to human immunodeficiency virus and hepatitis C virus infections?. Front Immunol.

[44] Kinder JM (2015). Cross-generational reproductive fitness enforced by microchimeric maternal cells. Cell.

[45] Goldman AS (1993). The immune system of human milk: antimicrobial, antiinflammatory and immunomodulating properties. Pediatr Infect Dis J.

[46] Hanson LA (1998). Breastfeeding provides passive and likely long-lasting active immunity. Ann Allergy Asthma Immunol.

[47] Kleist SA, Knoop KA (2020). Understanding the elements of maternal protection from systemic bacterial infections during early life. Nutrients.

[48] Victora CG (2016). Breastfeeding in the 21st century: epidemiology, mechanisms, and lifelong effect. Lancet.

[49] Ramanan D (2020). An immunologic mode of multigenerational transmission governs a gut Treg setpoint. Cell.

[50] Molès JP (2018). Breastmilk cell trafficking induces microchimerism-mediated immune system maturation in the infant. Pediatr Allergy Immunol.

[51] Goldman AS (1982). Immunologic factors in human milk during the first year of lactation. J Pediatr.

[52] Catassi C (1995). Intestinal permeability changes during the first month: effect of natural versus artificial feeding. J Pediatr Gastroenterol Nutr.

[53] Campbell DAJ (1984). Breast feeding and maternal-donor renal allografts. Possibly the original donor-specific transfusion. Transplantation.

[54] Gammill HS, Harrington WE (2017). Microchimerism: Defining and redefining the prepregnancy context - A review. Placenta.

[55] Beveridge NER (2007). Immunisation with BCG and recombinant MVA85A induces long-lasting, polyfunctional Mycobacterium tuberculosis-specific CD4^+^ memory T lymphocyte populations. Eur J Immunol.

[56] Darrah PA (2007). Multifunctional TH1 cells define a correlate of vaccine-mediated protection against Leishmania major. Nat Med.

[57] Ciuffreda D (2008). Polyfunctional HCV-specific T-cell responses are associated with effective control of HCV replication. Eur J Immunol.

[58] Rodrigue-Gervais IG (2010). Dendritic cell inhibition is connected to exhaustion of CD8^+^ T cell polyfunctionality during chronic hepatitis C virus infection. J Immunol.

[59] Precopio ML (2007). Immunization with vaccinia virus induces polyfunctional and phenotypically distinctive CD8(+) T cell responses. J Exp Med.

[60] Hesseling AC (2015). Immunogenicity of BCG in HIV-exposed and non-exposed infants following routine birth or delayed vaccination. Int J Tuberc Lung Dis.

[61] Jones CE (2015). The impact of HIV exposure and maternal Mycobacterium tuberculosis infection on infant immune responses to bacille Calmette-Guérin vaccination. AIDS.

[63] Mahtab S, Coetzee D (2017). Influence of HIV and other risk factors on tuberculosis. S Afr Med J.

[64] Malaba TR (2017). Antiretroviral therapy use during pregnancy and adverse birth outcomes in South African women. Int J Epidemiol.

[65] Wedi COO (2016). Perinatal outcomes associated with maternal HIV infection: a systematic review and meta-analysis. Lancet HIV.

[66] Chen JY (2012). Highly active antiretroviral therapy and adverse birth outcomes among HIV-infected women in Botswana. J Infect Dis.

[67] Li H (2020). Maternal HIV infection and risk of adverse pregnancy outcomes in Hunan province, China: A prospective cohort study. Medicine (Baltimore).

[69] Tchakoute CT (2018). Breastfeeding mitigates the effects of maternal HIV on infant infectious morbidity in the Option B+ era. AIDS.

[70] Lambert NC (2004). Quantification of maternal microchimerism by HLA-specific real-time polymerase chain reaction: studies of healthy women and women with scleroderma. Arthritis Rheum.

[71] Gammill HS (2010). Effect of parity on fetal and maternal microchimerism: interaction of grafts within a host?. Blood.

[72] Kanaan SB (2019). Immunogenicity of a rheumatoid arthritis protective sequence when acquired through microchimerism. Proc Natl Acad Sci U S A.

[73] Hanekom WA (2004). Novel application of a whole blood intracellular cytokine detection assay to quantitate specific T-cell frequency in field studies. J Immunol Methods.

[74] Guthrie KA (2016). Statistical methods for unusual count data: examples from studies of microchimerism. Am J Epidemiol.

[75] Kanaan SB (2021). Cord blood maternal microchimerism following unrelated cord blood transplantation. Bone Marrow Transplant.

